# Maxillary Intraosseous Plexiform Schwannoma: A Rare Neoplasm Radiographically Imitating an Odontogenic Lesion

**DOI:** 10.1007/s12105-025-01864-1

**Published:** 2025-11-14

**Authors:** Kittiphoj Tikkhanarak, Emily Lanzel, Sherry Timmons, John Hellstein, Thomas Heidenreich, Nidhi Handoo

**Affiliations:** 1 Department of Oral Pathology, Radiology and Medicine, College of Dentistry, The University of Iowa, Iowa City, IA USA; 2 Centrasota Oral & Maxillofacial Surgeons, Saint Cloud, MN USA

**Keywords:** Plexiform, Schwannoma, Neurilemmoma, Intraosseous, Maxilla, Jaw, Gnathic

## Abstract

Intraosseous plexiform schwannoma of the jaw is exceedingly rare, with only one previously reported case in the maxilla. We present a case of a 27-year-old male who presented with a painless maxillary swelling. Radiographic findings reveal an unilocular radiolucent lesion mimicking an odontogenic tumor or cyst. Histopathologic examination showed a multinodular spindle cell neoplasm showing Antoni A and B patterns and diffuse S100 positivity, confirming the diagnosis of intraosseous plexiform schwannoma. Although rare, this entity can mimic an odontogenic lesion on imaging. Awareness of this possibility is crucial to expand the differential diagnosis of radiolucencies of the jaw, and this case underscores the importance of histopathologic assessment for accurate diagnosis and appropriate clinical management.

A 27-year-old male presented with a painless swelling of unknown duration in the right maxilla. He was otherwise healthy and reported no significant medical history.

Cone beam computed tomography (CBCT) revealed a well-defined unilocular hypodense lesion measuring 1.8 × 1.5 × 1.1 cm in the right posterior maxilla, extending from the first molar to the canine region. The lesion exhibited buccal cortical expansion, cortical perforation, and root resorption of teeth #4 and #5 (Fig. 1).

**Fig. 1:**
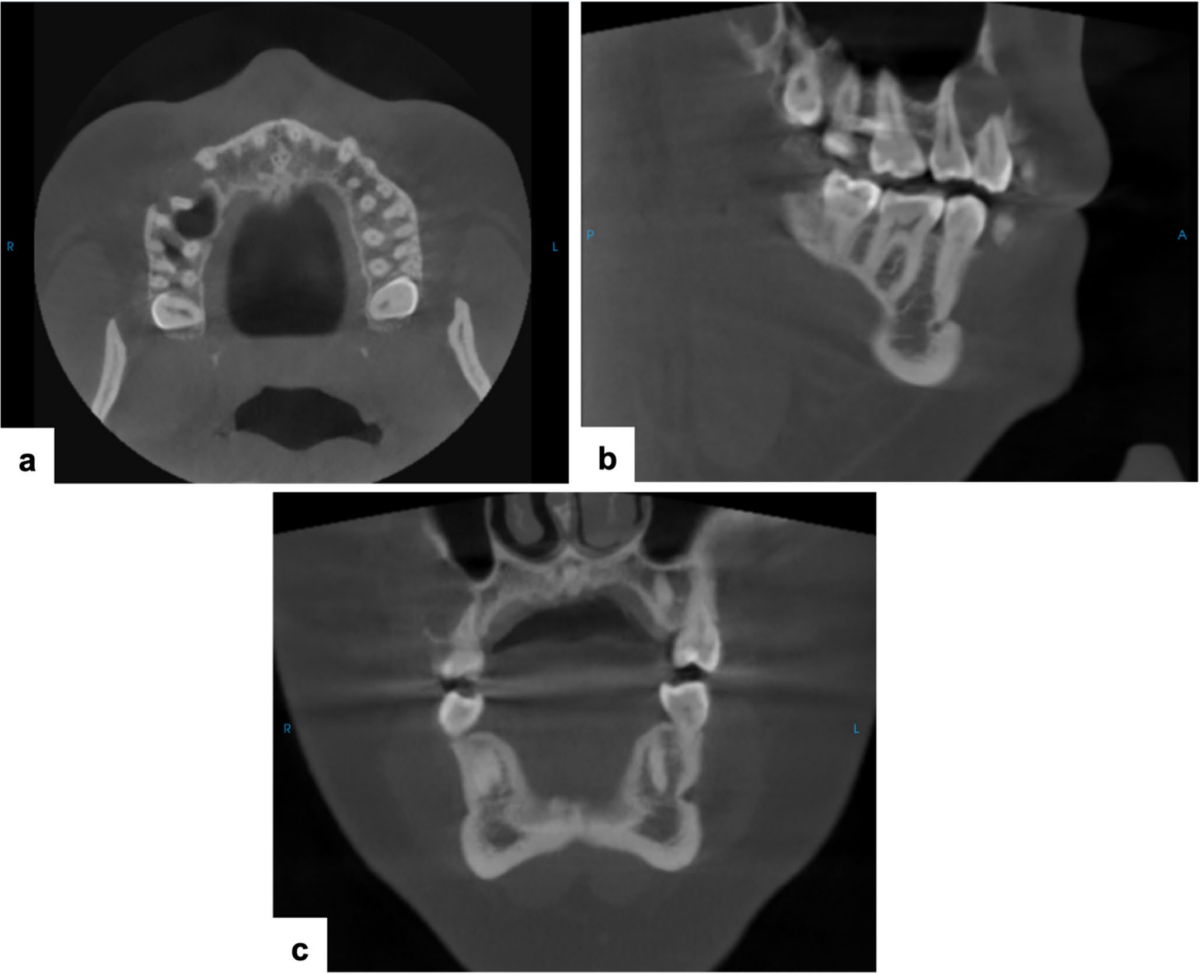
Cone beam computed tomography (CBCT) images. (**a**) Axial view: a well-defined unilocular hypodense lesion extending from extending from the mesial aspect of tooth #3 to the distal aspect of tooth #6, with buccal cortical expansion and focal perforation. (**b**) Sagittal view: root resorption of tooth #4. (**c**) Coronal view: buccal root resorption of tooth #5 and thinning of the maxillary sinus cortical wall without evidence of sinus involvement

Considering the patient’s age, clinical history, the site of lesion, and radiographic features, the most likely differential diagnosis included benign odontogenic cysts and tumors. With the high frequency of odontogenic keratocyst and ameloblastoma in gnathic bones ameloblastoma was a primary possibility based on the lesion’s aggressive radiographic features, however, other benign odontogenic tumors (e.g. ameloblastic fibroma and central odontogenic fibroma) could not be excluded in the differential. Less likely possibilities included benign non-odontogenic lesions such as central giant cell granuloma and other benign mesenchymal neoplasms.

The lesion was surgically enucleated and curetted, and the specimen was submitted to the Surgical Oral Pathology Laboratory at the University of Iowa College of Dentistry. Histopathologic examination showed a multinodular growth composed of well-circumscribed nodules surrounded by thin fibrous connective tissue (Fig. 2a, b). Within these nodules, there were areas of spindle-shaped tumor cells were arranged in a palisading pattern around acellular eosinophilic zones forming Verocay bodies (Fig. 2c); these features were characteristic of the Antoni A pattern. In other areas, the spindle-shaped cells were more loosely arranged within a myxoid stroma (Fig. 2d); these features correspond to the Antoni B pattern. Alternating Antoni A and Antoni B areas were observed in some areas (Fig. 2e). Immunohistochemistry demonstrated diffuse nuclear and cytoplasmic positivity for S100 (Fig. 3a, b) and diffuse nuclear positivity for SOX10 (Fig. 3c, d) in the lesional cells. EMA highlighted thin perineurial capsule surrounding individual tumor nodules (Fig. 3e, f). The morphologic and immunohistochemical findings supported the diagnosis of intraosseous plexiform schwannoma.

**Fig. 2:**
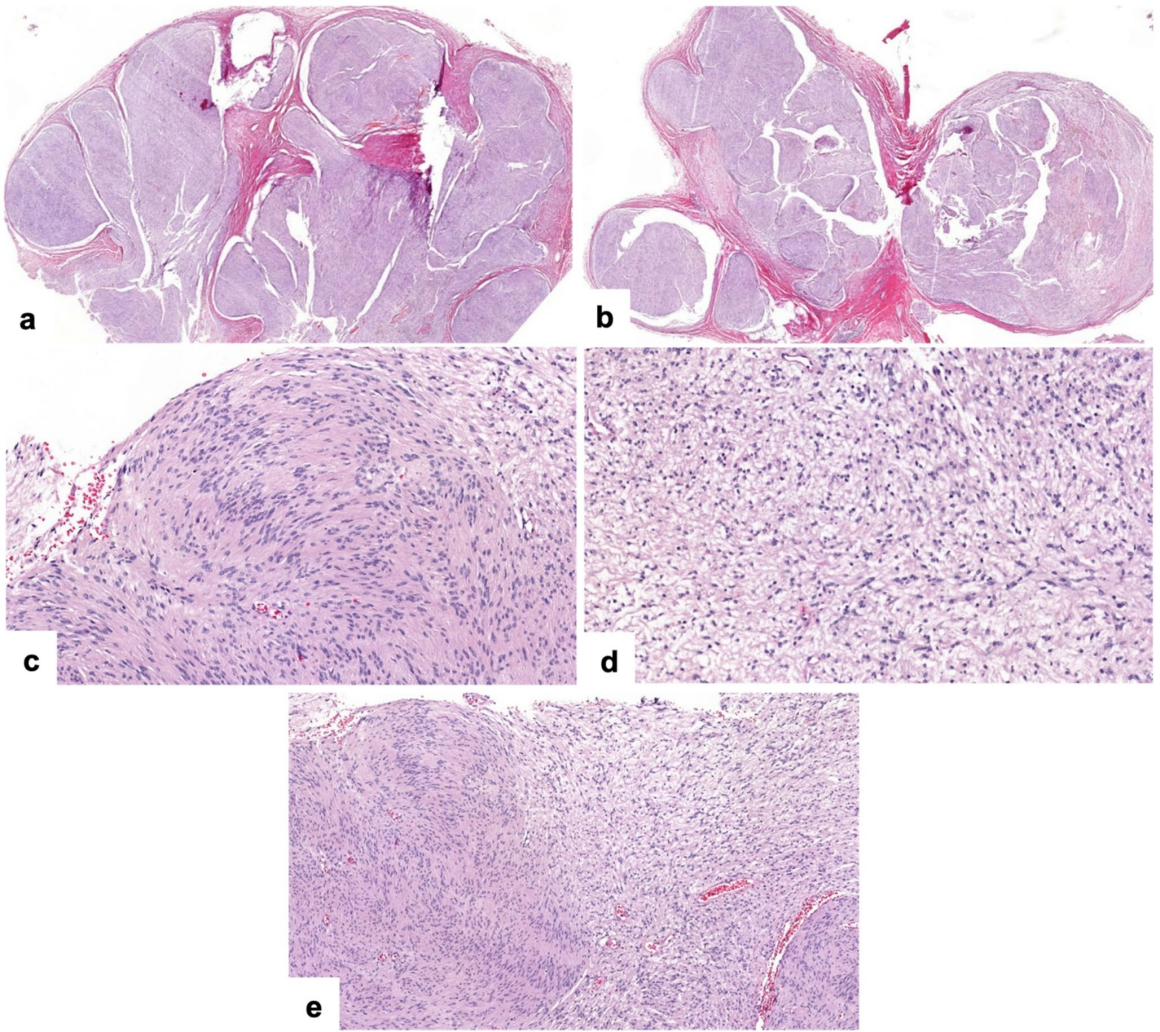
Histopathologic features. (**a**, **b)** Multilobular tumor nodules surrounded by fibrous connective tissue (hematoxylin and eosin [H&E], × 20). (**c)** Antoni A pattern: Spindle cells arranged in palisading with Verocay body formation (H&E, × 200). (**d)** Antoni B pattern: Loosely arranged spindle cells in a myxoid stroma (H&E, × 200). (**e)** Alternating Antoni A (left and lower right) and B (upper right) areas within the same nodule (H&E, × 100)

**Fig. 3:**
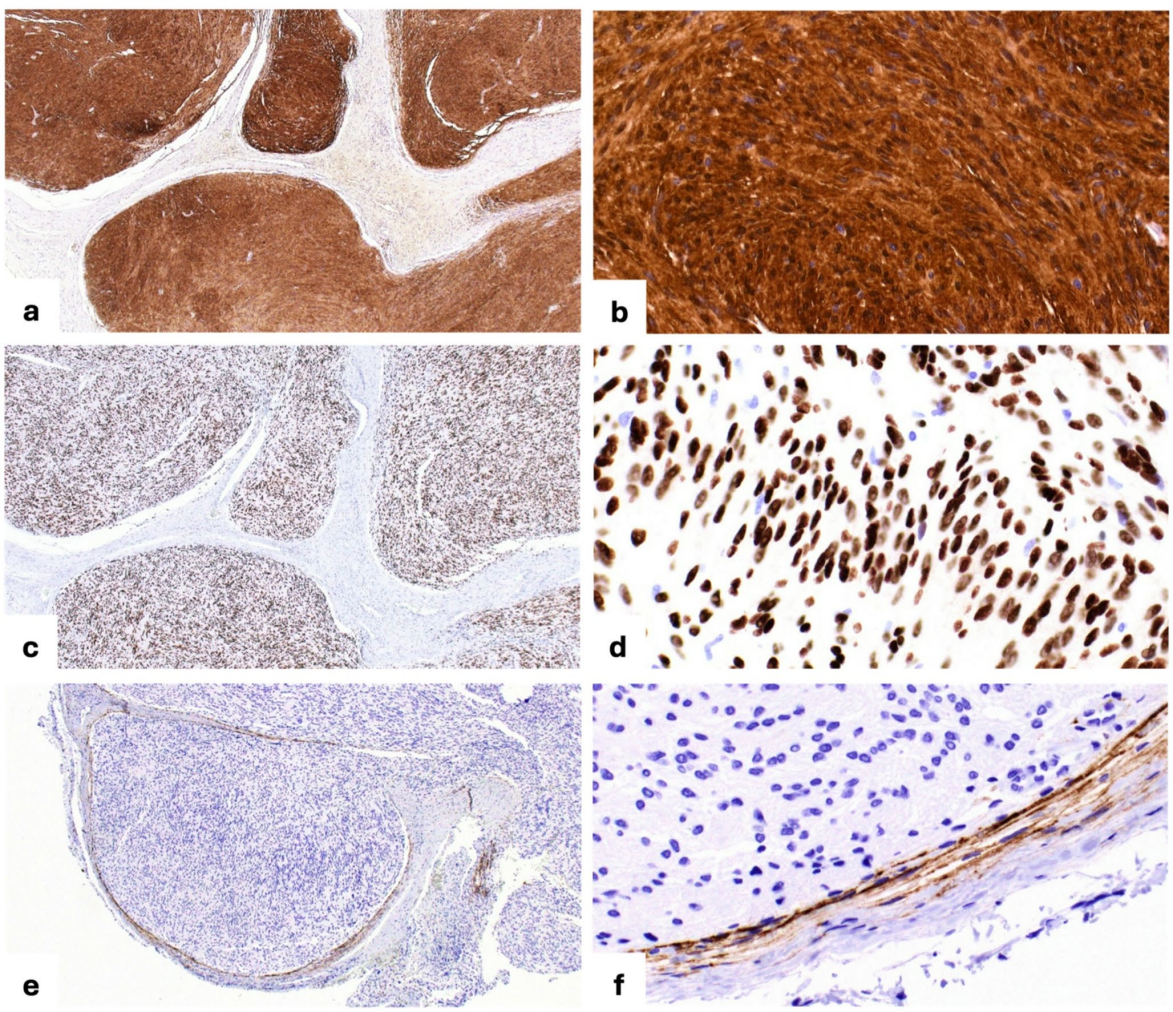
Immunohistochemical profile. (**a**, **b)** Diffuse nuclear and cytoplasmic immunoreactivity for S100 protein in lesional cells within tumor nodules (immunohistochemistry, × 40 and × 400). (**c**, **d)** Diffuse nuclear immunoreactivity for SOX10 protein in lesional cells within tumor nodules (immunohistochemistry, × 40 and × 400). (**e**, **f)** EMA highlighting thin perineurial capsule surrounding individual nodules (immunohistochemistry, × 40 and × 400)

Plexiform schwannoma is an uncommon histologic variant of schwannoma characterized by a multinodular architecture, most frequently composed of cellular (Antoni A) areas. It frequently presents in childhood and shows a predilection for the head and neck region and extremities [1]. Local recurrence has been reported more often in plexiform schwannomas than in classic variants, possibly due to their multinodular growth pattern, which may compromise complete surgical excision [2]. While most cases arise sporadically, occasional associations with neurofibromatosis type 2 (NF2) and schwannomatosis have been reported [1, 2]. In this case, the patient had reported no relevant medical or clinical history, supporting this as an isolated occurrence.

Intraosseous schwannomas of the gnathic bones are rare, with 111 cases reported in the literature to date—97 involving the mandible and only 14 affecting the maxilla [3–9]. Among these, the plexiform variant is exceedingly rare, with only four reported cases, none of which were associated with NF2 or schwannomatosis [6–9]. This variant represents approximately 3.7% of all reported gnathic schwannomas, with only one case occurring in the maxilla [9].

To our knowledge, the present case represents the second documented case of a plexiform schwannoma arising in the maxilla. Radiographically, intraosseous plexiform schwannomas in the jaws can resemble odontogenic lesions, making clinical and imaging-based diagnosis challenging. Therefore, this case illustrates the significance of a broad differential diagnosis list, biopsy and subsequent diagnosis based on histologic assessment. Accurate diagnosis allows for appropriate management including further investigation to rule out syndrome-related lesions.

## Data Availability

No datasets were generated or analysed during the current study.

## References

[1] WHO Classification of Tumours Editorial Board (2020) Soft tissue and bone tumours. 5th ed. WHO Classification of tumours series. International Agency for Research on Cancer, Lyon (France).

[2] Magro G, Broggi G, Angelico G, Puzzo L, Vecchio GM, Virzì V, Salvatorelli L, Ruggieri M (2022) Practical approach to histological diagnosis of peripheral nerve sheath tumors: an update. Diagnostics. 10.3390/diagnostics12061463 35741273 10.3390/diagnostics12061463 PMC9222088

[3] Peraza Labrador AJ, Valdez LHM, Marin NRG, Ibazetta KAR, Villacis M, Chacon JL, Huaman HO, Pariahuamán HC, Angulo HB, Woo V (2023) Gnathic schwannomas: a report of two cases and systematic review of the literature. Head Neck Pathol 17:984–999. 10.1007/s12105-023-01595-1 38055108 10.1007/s12105-023-01595-1 PMC10739683

[4] Vijayalakshmi KR, Jain V, Mehta S, Mamthashri V, Kavya BT (2024) Intraosseous schwannoma mimicking periapical radiolucency of mandible. Discov Med 1:21. 10.1007/s44337-024-00032-8

[5] Li S, Wang Z, Li Z, Xie S, Shan X, Cai Z (2024) Intraosseous schwannoma of the mandible: new case series, literature update, and proposal of a classification. Int J Oral Maxillofac Surg 53:205–211. 10.1016/j.ijom.2023.08.006 37716827 10.1016/j.ijom.2023.08.006

[6] Bhandarkar GP, Shetty KV, Desai D (2020) Intramandibular plexiform schwannoma presenting as a nonspecific benign lesion: how specific can we be? A case report with a review of the literature. J Cancer Res Ther 16:641–646. 10.4103/jcrt.JCRT_393_19 32719282 10.4103/jcrt.JCRT_393_19

[7] Bavle RM, Thambiah LJ, Nanda Kumar H, Srinath N, Paremala K, Sudhakara M (2011) Unusual intramandibular plexiform shwannoma. Oral Surg 4:51–55. 10.1111/j.1752-248X.2010.01111.x

[8] Vera-Sempere F, Vera-Sirera B (2010) Intraosseus plexiform schwannoma of the mandible: immunohistochemical differential diagnosis. J Craniofac Surg 21:1820–1824. 10.1097/SCS.0b013e3181f43f5b 21119430 10.1097/SCS.0b013e3181f43f5b

[9] Lambade PN, Lambade D, Saha TK, Dolas RS, Dhobley A (2013) Unusual intramaxillary plexiform schwannoma. Oral Maxillofac Surg 17:137–140. 10.1007/s10006-012-0345-6 22847037 10.1007/s10006-012-0345-6

